# Social group composition modulates the role of last male sperm precedence in post‐copulatory sexual selection

**DOI:** 10.1111/jeb.14191

**Published:** 2023-06-21

**Authors:** Juliano Morimoto, Grant C. McDonald, Stuart Wigby

**Affiliations:** ^1^ School of Biological Sciences University of Aberdeen Aberdeen UK; ^2^ Programa de Pós‐graduação em Ecologia e Conservação Universidade Federal do Paraná Curitiba Brazil; ^3^ Institute of Mathematics University of Aberdeen, King's College Aberdeen UK; ^4^ Department of Ecology University of Veterinary Medicine Budapest Budapest Hungary; ^5^ Department of Evolution, Ecology, and Behaviour, Institute of Infection, Veterinary & Ecological Sciences University of Liverpool Liverpool UK

**Keywords:** behavioural ecology, density‐dependent, polyandry, sexual conflict, sperm competition

## Abstract

In many species, the order in which males mate with a female explains much of the variation in paternity arising from post‐copulatory sexual selection. Research in *Drosophila* suggests that mating order may account for the majority of the variance in male reproductive success. However, the effects of mating order on paternity bias might not be static but could potentially vary with social or environmental factors. To test this idea, we used an existing dataset, collated from an experiment we previously published (Morimoto et al., *PLoS One*, *11*, 2016, e0154468), with the addition of unpublished data from the same experiment. These previous experiments manipulated larval density in *Drosophila melanogaster* which generated variation in male and female body size, assembled groups of individuals of different sizes, and measured the mating success and paternity share of focal males. The data presented here provides information on each focal male's mating order and the frequency in which focal males remated with same females (‘repetitive matings’). We combined this information with our previously reported focal male reproductive success to partition variance in paternity into male mating order and repetitive matings across groups that differed in the body size composition of males and females. We found, as expected, that male mating order explained a considerable portion of the variance in male paternity. However, we also found that the impact of male mating order on male paternity was influenced by the body size composition of groups. Specifically, males that tended to mate last had a greater paternity advantage, and displayed lower variance, in groups containing a heterogenous mixture male body sizes than in groups with a single male body size. Repetitive mating only had a minor contribution to the variance in male paternity share across all experiments. Overall, our findings contribute to the growing body of research showing that post‐copulatory sexual selection is subject to socio‐ecological influences.

## Graphical abstract

Social group composition affects the role of last male sperm precedence in Drosophila.

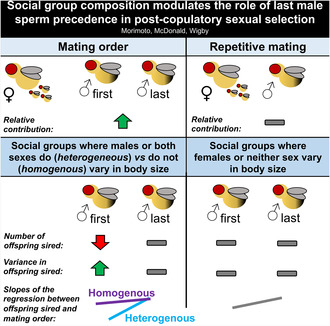

## 1 INTRODUCTION

Polyandry commonly allows sexual selection to continue after copulation, via sperm competition and cryptic female choice (Birkhead & Pizzari, 2002; Boulton & Shuker, 2013; Kvarnemo & Simmons, 2013; McDonald & Pizzari, 2018). Post‐copulatory sexual selection commonly results in mixed paternity, whereby multiple males sire offspring produced by a female in a single bout of reproduction (Birkhead & Pizzari, 2002). In many species, the order in which males mate with a female affects their relative paternity share (Birkhead & Hunter, 1990). For example, in many insects, the last male to mate with a female has a paternity advantage, known as last male sperm precedence (Gwynne, 1984). Previous work in *Drosophila melanogaster*, has demonstrated the presence of strong last male sperm precedence (Lefevre & Jonsson, 1962) that explains nearly all of the variance in paternity share, with little residual variance explained by other factors (Pischedda & Rice, 2012). Moreover, last male sperm precedence is shared between closely related *Drosophila* species highlighting its broader significance to post‐copulatory sexual selection (Manier et al., 2013). However, the majority of empirical investigations of last male sperm precedence in *Drosophila* have typically focused on experimental designs that allow females to copulate with only two males (see Laturney et al., 2018 and the references therein). Studies in nature, and in freely interacting social groups under laboratory conditions, indicate that female *Drosophila* may regularly mate with more than two sexual partners (see Laturney et al., 2018 and the references therein). Laturney et al. (2018) used fluorescent sperm markers to show that the length of the interval between matings (i.e., remating latency) in twice mated females had no relationship with last male sperm precedence, whereas in thrice mated females, shorter remating latencies were associated with reduced last male sperm precedence. These results suggests that paternity biases emerging from last male sperm precedence in more realistic social groups may be more complex than originally predicted.

In complex polyandrous social groups, variation in male and female genotypes, age and condition may all contribute to patterns of sperm competition and paternity, including the strength of biases in paternity due to last male sperm precedence (Amitin & Pitnick, 2007; Chow et al., 2010; Clark et al., 1995; De Nardo et al., 2021; Fiumera et al., 2005, 2007; Lüpold et al., 2011, 2012, 2013, 2020; Macartney et al., 2019; Reinhart et al., 2014; Sepil et al., 2020; Vega‐Trejo et al., 2019). Importantly, many morphological (e.g., sperm length) and behavioural (e.g., copulation duration) traits related to paternity biases from last male sperm precedence appear to be shared among closely related *Drosophila* species (Manier et al., 2013), suggesting that last male sperm precedence is an evolutionary response to male mating context. Males of many species can also adjust their relative ejaculate investment and mating behaviour in response to their past or present social environment, which can influence paternity outcomes (Bretman et al., 2011; Dore et al., 2020; Perry et al., 2013; Price et al., 2012; Wedell et al., 2002; Wigby et al., 2009). For example, males can mate more than once with the same female (‘repetitive mating’), an effect that can increase the representation of the male's sperm and hence typically leads to higher paternity (Birkhead & Montgomerie, 2020; Carleial et al., 2020; Harts et al., 2016; Morimoto, McDonald, et al., 2019). While repetitive mating is not equivalent to last male sperm precedence sensu stricto, it can increase the chances that males mate in the last male position while also increasing the proportion of sperm allocated to the female, thereby increasing males' overall chances of siring higher proportions of the offspring. In *D. melanogaster*, we know that last male sperm precedence is influenced by multiple factors such as remating timing and polyandry levels (Laturney et al., 2018), female age (Mack et al., 2003), and male condition (De Nardo et al., 2021). For example, males that experienced dietary protein restriction and have small body sizes achieve higher paternity share and displace more competitor sperm compared with larger normally fed males (De Nardo et al., 2021). Moreover, repetitive mating is favoured in highly polyandrous mating groups as a way for males to increase paternity (Morimoto, McDonald, et al., 2019). However, given past studies have commonly evaluated paternity biases in experimental scenarios where females are limited to only two mating partners, we lack information on how within group variation in such male and female characteristics impact on the role of last male sperm precedence and the relationship between male mating order and paternity share in freely mating populations.

**FIGURE 1:**
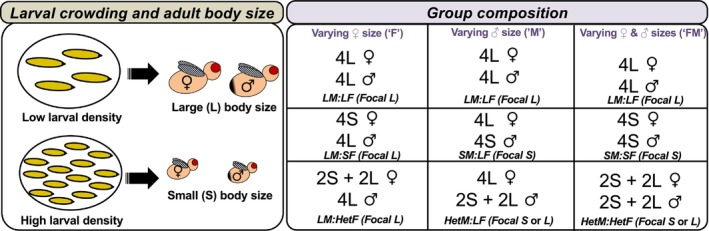
Schematic representation of the experimental design. We manipulated larval density to generate flies with small and large body sizes of both sexes. Next, we assembled replicate vials with four flies of each sex (i.e., eight flies per vial in total), in three different (and independent) experiments: (1) Varying female size, (2) Varying male size, and (3) Varying male and female sizes.

**FIGURE 2:**
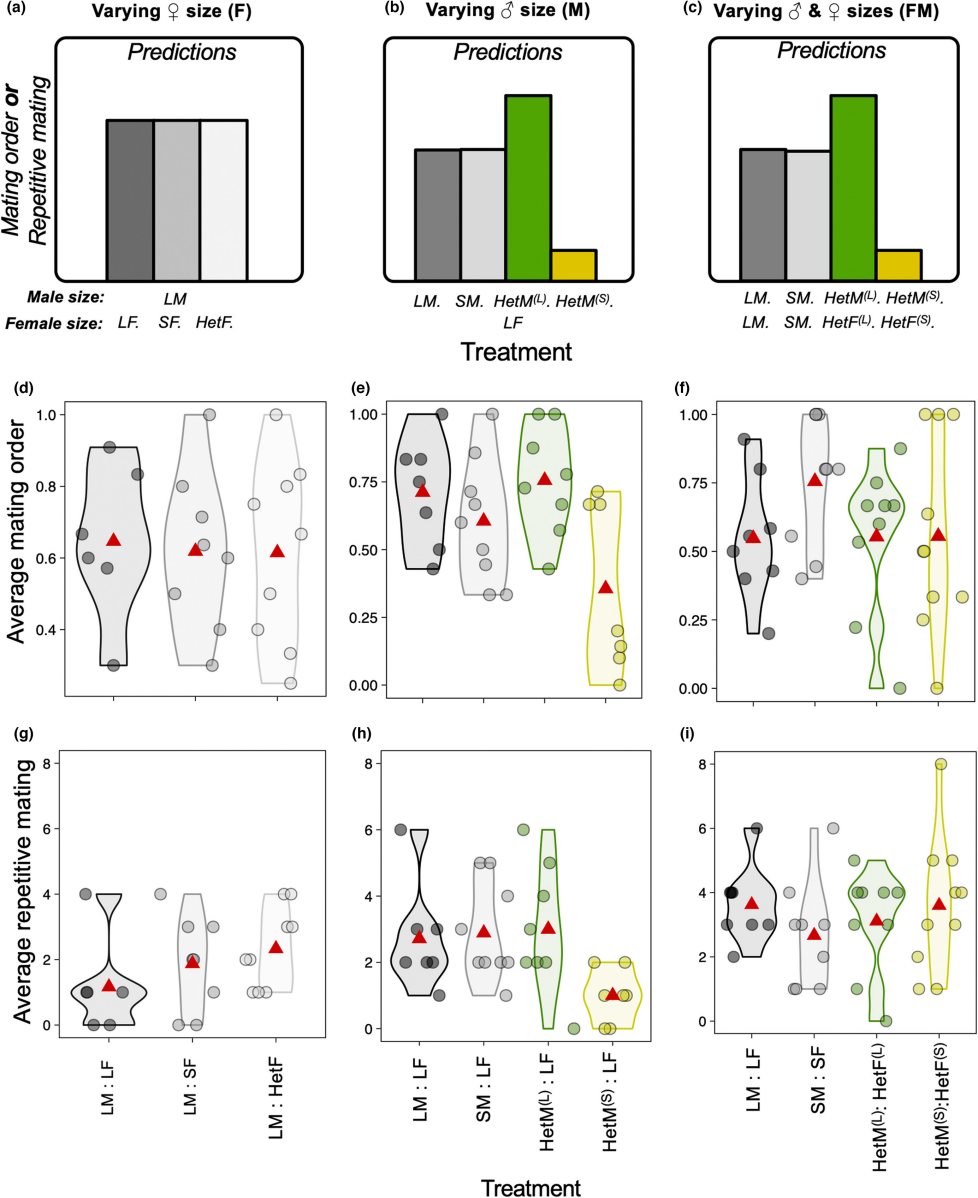
The effects of body size and social group composition on focal male mating order and repetitive matings. (a–c) Predictions for the observed traits for the focal males measured across treatments. (d–f) Average male mating order for each focal male, a proxy of focal male mating position in the queue of females' mates. (g–i) Average number of repetitive mating with same females for each focal male across experiments. *Hom*: Homogenous group in terms of size of the sex(es) being manipulated. *Het*: Heterogenous group in terms of size of the sex(es) being manipulated. *L*: Large individuals of the sex(es) being manipulated. *S*: Small individuals of the sex(es) being manipulated. Superscripts: *F*: experiment varying female size. *M*: experiment varying male size. *FM*: experiment varying female and male sizes. Red triangles mark the grand average value across all males in each treatment.

**FIGURE 3:**
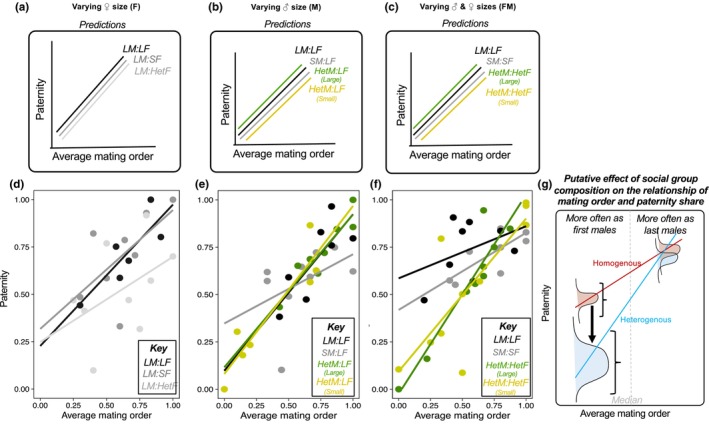
The effects of body size and social groups composition on the relationship between focal male mating order and paternity. (a–c) Predictions. We had no *a piori* expectation that the relationships would differ for focal males across different groups. Note that, for visualization purposes, we drew lines adjacent for each focal male treatment, but had no reason to expect intercepts to differ. (d–f) Empirical gradients (i.e., slope) of the relationships between average focal male mating order and focal males' paternity across experiments. (g) Schematic representation of the decrease in average and increase in variance in paternity share for focal males mating more often as first male in Het groups (i.e., experiments varying male and female and male sizes). This is the underpinning mechanism by which the slope of male mating order on paternity is steeper in Het in the experiment varying female and male sizes and to a smaller extent, in the experiment where male varied in size. *Hom*: Homogenous group in terms of size of the sex(es) being manipulated. *Het*: Heterogenous group in terms of size of the sex(es) being manipulated. *L*: Large individuals of the sex(es) being manipulated. *S*: Small individuals of the sex(es) being manipulated. Superscripts: *F*: experiment varying female size. *M*: experiment varying male size. *FM*: experiment varying female and male sizes.

Here, we address this gap in knowledge by using replicate freely mating polyandrous groups of *D. melanogaster* to investigate the effects of larval density, and the resulting changes in male and female adult body size, on patterns of male paternity share within multiply mated females. Specifically, we aimed to investigate how the body size composition of social groups impacts on (i) male mating order and the frequency of repetitive matings between males and their female partners, (ii) the relative contribution of mating order and repetitive matings to variance in male paternity share and (iii) the relationship between male mating order and male paternity share. To address these objectives, we present new analyses on an existing dataset, collated from an experiment we previously published (Morimoto et al., 2016), with the addition of previously unpublished data. The original experiments manipulated larval density in *D*. *melanogaster* to generate males and females that had small and large body sizes, respectively (Figure 1; Morimoto et al., 2016). While larval density manipulation affects multiple traits (e.g., body size, remating rate, female fecundity, mating success [Bretman et al., 2016; Than et al., 2020]), we henceforth refer to the two body size classes (large and small) as a shorthand for low and high larval density, respectively. After manipulating body size, we assembled groups with four males and four females to test for the influence of the body size composition of social groups on male paternity. One male and all females in the group had a phenotypic marker (*sparkling* eyes) while the remaining males were wild‐type (rival males). This approach allowed us to assign paternity of the focal male. We varied female size (experiment 1), male size (experiment 2), or both (experiment 3; see Figure 1), and tracked the paternity share of the (large or small) focal male across the experimental groups. We used data from our previously published study that focused on mating observations and paternity shares (Morimoto et al., 2016), and extracted data on the patterns of male mating order and repetitive mating with females, in order to partition the variance in male paternity share with respect to these latter two factors. This approach allows us to examine to what extent the strong mating order effect found by Pischedda and Rice (2012) and others in the field (Morrow et al., 2005; Schnakenberg et al., 2012) holds up under varying social conditions, or if varying social group composition in terms of body size impacts on the role of last male precedence and male mating order on paternity. More broadly, this approach allowed us to expand our understanding of how the social groups composition may influence post‐copulatory sexual selection. Note that the ‘re‐analysis of previous data’ approach has been used in other studies in the insect literature to gain further insights into complex datasets (Morimoto, 2022; Vahl et al., 2013). Our predictions are as follows:

1. We predicted the male to mate in the last position should sire a higher proportion of female's offspring (i.e., higher paternity share) due to last male sperm precedence (Pischedda & Rice, 2012), and thus we further predicted that mating order would explain much of the variation in paternity share across all contexts.
2. Previous studies indicate no effect of female size on last male sperm precedence (see, e.g., figure 4 in Amitin & Pitnick, 2007). We therefore predicted that, in experiments where female size was manipulated, female size would have no effect on the relationship between male mating order and male paternity.
3. Based on previous studies (Friberg & Arnqvist, 2003; Long et al., 2009; Morimoto et al., 2016), we predicted that male size impacts patterns of pre‐copulatory competition such that large males have higher mating success. Specifically, when focal male sizes varied, we expected large focal males to have higher mating frequency and consequently, repetitive mating with same female by two mechanisms (see Predictions in Figures 2 and 3). First, we expected large focal males to overcome female resistance more successfully, and thus gain higher number of copulations than small focal males (Long et al., 2009; Morimoto et al., 2016; Pitnick & García‐González, 2002). Second, when large focal males are competing directly with small focal males for access to females, we expected large males to win a higher proportion of copulations, and relatively more copulations as last male (Morimoto et al., 2016; Pitnick & García‐González, 2002). We expected both of these effects to be particularly apparent when females were previously mated because non‐virgin females have heightened resistance to mating (Manning, 1962). As a result, we expected large focal males to have higher average paternity share relative to small focal males with multiply mated females due to both greater overall mating frequency and repetitive mating, as well as the benefits of last male precedence arising from mating more often in the last position.
4. We predicted male mating order to show a correlation with male paternity share with a steeper slope as compared to any correlation between repetitive mating and paternity share (Pischedda & Rice, 2012). Recent evidence suggests that small males should achieve higher paternity with twice‐mated females (De Nardo et al., 2021), although this effect was not observed in freely mating groups (Morimoto et al., 2016). Therefore, we did not have enough a priori information to formulate specific predictions on the impact of body size manipulations on the effects of mating order on paternity share in freely mating populations.

## 2 MATERIALS AND METHODS

Detailed methods of the fly experiments have been described in Morimoto et al. (2016) (see also Figure 1). Here, we provide a brief overview of the experimental design and detail the additional data and analysis approach adopted for this paper.

### 2.1 Summary of fly experiments

Focal males were *wild‐type* Dahomey, while rival males and experimental females carried the recessive *sparkling* ^*poliert*^ mutation (*spa*) backcrossed into Dahomey. We raised flies in two different larval densities: high density ~100 larvae/mL of food and low density ~ four larvae/mL of food. Flies were collected as virgins and housed in single sex groups of 15–20 individuals for 2–5 days (i.e., flies were 2–5 days old at the onset of experiments). Before experiments, all flies were colour marked with acrylic paint under light ice‐anaesthesia 3 h prior to the start of the experiment, and allowed to recover in same sex and colour vials for 2 h. This allowed us to identify and track individuals throughout the experiment (Tan et al., 2012). We opted for ice anaesthesia to minimize the negative confounding effects of CO_2_ on fly mating behaviour (Barron, 2000), but we cannot rule out any potential confounding effects of ice anaesthesia in small and large individuals. We assembled replicate vials with four flies of each sex (i.e., eight flies per vial) using an aspirator without anaesthetising the flies. In each vial, all females and three males had the recessive eye phenotype mutation *sparkling* ^*poliert*^ (henceforth “*spa*”), while one focal male was *wild‐type*. This allowed us to calculate focal male paternity based on the eye phenotype of the offspring. Males and females were allowed to interact for 4 h per day, where all matings were recorded, for four consecutive days; females were (individually) allowed to lay eggs for 20 h in between the interaction periods while males were kept in groups. The same individuals were gathered into the same groups for the consecutive days of interactions. All flies were discarded on the fifth day. As our low larval density generates the largest individuals ([Morimoto et al., 2016, 2017], as is the case in other flies [Blanckenhorn, 1998; Lyimo et al., 1992; Morimoto, Nguyen, et al., 2019; Stockley & Seal, 2001]), we used individuals of the low larval density treatment as the reference treatment. For simplicity, we henceforth refer to flies raised at low larval density as ‘large’ flies, and flies raised at high larval density ‘small’ flies. We performed three independent experiments. The first experiment focuses on varying the sizes of females in the group, the second focuses on varying the sizes of males in the group, and the third, on varying the size of both sexes simultaneously:

1. Varying female size, where groups of either large and small females, or a mix of both sizes, were assembled with large males. Focal males always competed against three non‐focal males of the same (large) size (Figure 1).
2. Varying male size, where groups of either large or small males, or a mix of both sizes, were assembled with large females. Focal males (either large or small) competed either with three focal males of the same size, or one rival male of the same size as the focal male and two non‐focal males of the other size (Figure 1).
3. Varying male and female size, where groups of large or small males and females, or a mix of both sizes were assembled. Focal males competed as in the ‘Varying male size’ experiment, with the addition that females also varied in sizes (Figure 1).

Each experiment was conducted independently. For focal males in each experiment the size of their competitors and potential mates differed in terms of the sex under manipulation (female, male, or both). Henceforth, we refer to homogenous when all individuals of a given sex being manipulated had the same size within groups (i.e., from the same larval density), while heterogenous refers to when individuals of a given sex being manipulated varied in size within groups and included a mix of large and small individuals. We thus use the terminology of ‘Hom’ and ‘Het’ to signal whether all individuals of a given sex within groups were homogenous or heterogeneous in body size, and the letters ‘F’, ‘M’, and ‘FM’ to indicate which of the sexes—female, male or both male and female respectively—were being manipulated. We therefore use the following terminology, where the size of the focal male is given in brackets:

### 2.2 Varying female size (F)

1. LM:LF (Focal L): for the experiment where all males and females were large.
2. LM:SF (Focal L): for experiments where all males were large and all females were small.
3. LM:HetF (Focal L): for experiments where all males were large and females were large and small.

### 2.3 Varying male size (M)

1. LM:LF (Focal L): for the experiment where all males and females were large.
2. SM:LF (Focal S): for experiments males were small and all females were large.
3. HetM:LF (Focal S or L): for experiments where males were large and small, and all females were large. Focal males were either small or large.

### 2.4 Varying female and male size (FM)

1. LM:LF (Focal L): for the experiment where all males and females were large.
2. SM:SF (Focal S): for experiments where all males and females were small.
3. HetM:HetF (Focal S or L): for experiments where the sizes of both males and females varied. Focal males were either small or large.

Figure 1 summarizes our experimental design and terminology.

### 2.5 Data analyses

#### 2.5.1 Paternity

There were 20 replicate groups for Het and 10 for Hom groups for each of F, M and FM manipulations (i.e., *n* = 40 vials for each of F, M and FM, 120 in total). For HetM and HetFM, we measured 10 groups with small and 10 groups with large focal males, making our observations of focal males of different sizes independent. For each focal male size, we calculated focal male paternity share (PS) as:

$$\mathrm{PS}=\sum_{i=1}^{n}\frac{\mathrm{wt}\;\text{offspring}}{\text{total offspring}}$$

where wt is the number of wild‐type offspring, for all females (*i*‐*n*) that mated with the focal male and at least one rival male (i.e., females that mated with ≥2 males, with one male being the focal male). Thus, paternity share was calculated only on conditions when sperm competition was possible. For example, if a female mated with a focal male on day one and with a rival male on day three, we only consider focal male paternity share of the offspring from days three and four, when direct sperm competition could occur.

#### 2.5.2 Mating order and repetitive matings

We calculated the proportion of mating events in which focal males were the last of ≥2 males to mate with a female (i.e., male mating order) and the number of focal male repetitive matings (i.e., the sum of the number of matings a focal male had with each of his female mating partners that also mated with other rival males). We calculated focal male mating order, using the approach of Pischedda and Rice (2012), as follows: for each female on each day, we assigned the value of ‘1’ if the focal male was the last to mate with that female (or ‘0’ if the focal male was not the last to mate with that female). We then calculated the average focal male mating order as the average score across all females the focal male mated with, and across all days of the experiment (i.e., average closer to 1 means more in the last male role with all mates, and closer to zero means more in an earlier male role with all mates). Note that even if females stopped mating before the end of the four days of interactions and after mating with two males (one being the focal), females still contributed to an index of 0 or 1 for subsequent days of the experiment for the focal male mating order. For example, if females mated with a rival male on day 1 and then the focal male in day 2, then stopped mating for the remaining days of the experiment, the index for the focal male was scored as a ‘1’ for days 2, 3, and 4, indicating he was in the last male position for these days. This methodology was adopted in agreement with the general approach proposed by Pischedda and Rice (2012) and ensures that an index of mating order was assigned to each of the focal males' mates while sperm competition could occur. We did not have individual paternity data for rival males due to our experimental design in which all rivals shared the same *spa* phenotypic marker (see above), and thus only focal paternity could be measured.

#### 2.5.3 Statistical analysis

We analysed each experiment separately as they were conducted independently. First, we fitted models with social group composition (i.e., Het vs. Hom) and focal male size (small or large), and their interaction, on male mating order and repetitive matings. Note that the terminology Het and Hom in the models refer to the heterogenous or homogenous body sizes of females (experiment 1), males (experiment 2) or both (experiment 3). We used generalized linear models (GLMs) with *Poisson* error distribution for the analysis of repetitive matings and GLM with *Binomial* error distribution for mating order. Both models used *quasi* extension to account for overdispersion. We controlled for average group fecundity in all models by adding this as a covariate in all models (i.e., the average offspring production of all females in the vial). Student–Newman–Keuls (SNK) post‐hoc test from the ‘agricolae’ package (Mendiburu & Simon, 2015) was used to assess differences in focal male mating order and repetitive matings as a result of focal male group composition (Het vs. Hom) and size, whenever the *p*‐value of the *F*‐test was below the cut‐off value of 0.07 (i.e., marginally non‐significant and significant effects). We performed model selection using AIC criteria according to the ‘step’ function with default arguments to increase power and interpretability of the models and reported the results of the final model in the results section. Models with the lowest AIC scores were considered the final model. Assortative mating by body size was calculated as the number of matings between large and small focal male and females of the same body size in homogeneous and heterogenous environments. We used ANOVA to test for assortative matings.

Next, we decomposed the variance in male paternity share to investigate the relative contributions of mating order and repetitive matings, adapted from Pischedda and Rice's (2012) approach. To partition variance in male paternity share, we first fitted a general linear model using the ‘lm’ function in R which consisted of paternity share as a function of male mating order and repetitive matings while controlling for the effects of males' mate fecundity. There was no statistically significant correlation between mating order and repetitive matings which suggest no collinearity was present in our models by having the two covariates (Table S1). Mating order and repetitive matings were standardized (i.e., mean subtracted and divided by the standard deviation) prior to the analysis of relative contributions to control for any potential effects of absolute values in the Commonality Analysis (CA) partitioning. CA has been proposed as a method in ecological modelling to decompose the variances in multiple regression models (Ray‐Mukherjee et al., 2014). We performed CA using the ‘regr’ function of the ‘yhat’ package (Nimon & Roberts, 2009), which partitions the explained variance explained by the regression model in terms of the relative contributions of standardized mating order and standardized mate repetitive matings.

We then investigated the putative effects of social group composition on the relationship between the average focal male mating order and repetitive matings on focal male paternity share. In the experiment varying female size, we fitted two linear models each with a two‐way interaction of either standardized mating order or standardized repetitive matings with focal male treatment (i.e., whether focal males were with large only [HomF] or both small and large females [HetF]). This allowed us to test the differential effects of female size on the contributions of mating order and repetitive matings to focal male paternity. In the experiment varying male size, we fitted a similar model, but the interactions investigated how focal male size (Small vs. Large) and social group composition (i.e., Hom vs. Het) interacted with mating order and repetitive matings to influence focal male paternity share. Lastly, in the experiment varying both male and female sizes, the interactions tested how the combination of focal male size (Small vs. Large) and social group composition (Het vs. Hom) interacted with mating order and repetitive mating to affect focal male paternity share. All analyses were performed in R 3.2.2 (R Core Team, 2019).

## 3 RESULTS

### 3.1 Small males had fewer matings in the last male position in groups with heterogenous body sizes

In the experiments varying female size and experiments varying female and male sizes, there was no difference between Het and Hom groups in male mating order index (the average position of the focal male in the mating queue; Table S2; Figure 2d,f). In the experiment varying male size, our results show male size affected male mating order index as predicted (*F*_1,27_ = 7.991, *p* = 0.008, Table S2), whereby the average mating order index of small focal males was significantly lower than that of large focal males (i.e., large males tended to mate on average more in the last male position, Figure 2e). Large focal males remated relatively more than small focal males in the Het versus Hom groups (*Social* × *Size*: *F*_1,26_ = 6.62, *p* = 0.016, Table S2), an effect that was absent in the experiment varying female–male sizes (*Social × Size*: *F*_1,31_ = 1.178, *p* = 0.286, Table S2, Figure 2g–i). In the experiment varying female size, female sizes in the Het versus Hom groups had no effect on focal male repetitive mating (*Social × Size*: *F*_2,19_ = 0.726, *p* = 0.496). This suggests that, as expected, large males outcompeted small males when females had previously mated. That small males were more likely to experience lower mating order and repetitive mating frequency compared with large males only when females were large, but not when females also varied in size, potentially suggests that these effects are both context‐ and size‐dependent (Figure 2e,f and h,i). Importantly, there was no statistical evidence to suggest assortative mating when female size varied (*Female Size*: *F*_1,8_ = 0.285, *p* = 0.607). Likewise, there was no evidence of assortative mating between large focal males and large females compared with small focal males and large females when both female and male size varied (*Female Size × Focal Male*: *F*_1,30_ = 0.360, *p* = 0.552).

### 3.2 Male mating order on average contributed to more than 80% of total explained variance in male paternity

Although both male mating order and repetitive matings of focal males with the same female were size‐ and socially dependent (see above), each of these factors could have affected male paternity share in different ways. We therefore partitioned the variance in male paternity share to investigate the contributions of mating order and repetitive mating (Pischedda & Rice, 2012). We found that on average, male mating order accounted for >80% of the explained variance in paternity share across multiply mated females, whereas repetitive mating only accounted for <10% of explained variance in paternity across experiments (Table 1a). The high percentage of variance explained by male mating order was consistent across Hom and Het groups as well as between small and large males across all three experiments (i.e., it was socially and size‐independent). There was only one exception, where repetitive matings with same females contributed a sizable percentage (27.26%) of the explained variance in paternity: the homogenous treatment in the experiment varying male sizes where all males and females were large (Table 1b).

**TABLE 1:** Commonality analysis of male paternity, partitioning the explained variance into the contributions of male mating order (last male sperm precedence) and repetitive matings. (a) Overall analysis of variance portioning across all treatments within each experiment per focal male size. (b) Analysis of variance portioning per treatment and focal male size.

(a) Experiment	Female size	Male size	Focal male size	Explained variance in paternity	*of which* Mating order	%	Repetitive mating	%	Remaining	%
Varying female size	Variable	Constant	Large	0.499	0.446	89.33	0.045	9.12	0.008	1.55
Varying male size	Constant	Variable	Large/Small	0.665	0.584	87.90	0.001	0.15	0.069	11.95
Varying female and male sizes	Variable	Variable	Large/Small	0.583	0.488	83.60	0.005	0.08	0.090	16.32
**(b)**										
***per treatment***										
	Homogeneous	Homogenous	Large	0.998	0.235	23.68	0.005	0.59	0.758	75.73
	Large	Large								
Varying female size (F)	Homogeneous	Homogeneous	Large	0.537	0.208	38.91	0.011	2.05	0.318	59.04
	Small	Large								
	Heterogeneous	Homogeneous	Large	0.876	0.175	20.06	0.058	6.72	0.643	73.22
		Large								
	Homogenous	Homogenous	Large	0.933	0.129	13.86	0.254	27.26	0.55	58.88
Varying male size (M)	Large	Large								
	Homogenous	Homogenous	Small	0.619	0.182	29.51	0.003	0.52	0.434	69.97
	Large	Small								
	Homogenous	Heterogeneous	Large	0.938	0.253	27.03	0.006	0.68	0.679	72.29
	Large									
	Homogenous	Heterogeneous	Small	0.947	0.864	91.21	0.000	0.01	0.083	8.78
	Large									
	Homogenous	Homogenous	Large	0.789	0.191	24.51	0.004	0.55	0.594	74.94
Varying female and male sizes (FM)	Large	Large								
	Homogenous	Homogenous	Small	0.888	0.421	47.39	0.052	5.93	0.415	46.68
	Small	High								
	Heterogeneous	Heterogeneous	Large	0.884	0.773	87.37	0.000	0.02	0.111	12.61
	Heterogeneous	Heterogeneous	Small	0.733	0.702	95.76	0.025	3.48	0.006	0.76

### 3.3 Heterogenous body size composition in groups strengthened the association between male mating order and paternity

Since male mating order was the primary contributor to variance in paternity share of multiply mated females, we expected a non‐zero positive relationship between focal male mating order and paternity (Figure 3a–c). In line with this we identified a statistically significant main effect of focal male mating order on paternity share in all three experiments (*Varying female size*: *F*_1,19_ = 10.546, *p* = 0.004; *Varying male size*: *F*_1,27_ = 69.444, *p* < 0.001; *Varying male and female sizes*: *F*_1,32_ = 62.733, *p* < 0.001; Table S3), such that males with higher mating order indices (more often in the last position) sired a greater proportion of offspring under sperm competition conditions (Figure 3d–f). In the experiment varying male size, our top model included the interaction between treatment (Hom vs. Het) and focal male mating order even though it did not reach statistical significance (*p* > 0.05). The lack of statistical significance is likely because of the trend for a shallower slope identified for small males in Hom groups (Figure 2e). Nevertheless, the retention of this interaction means that the relationship between focal male mating order and paternity was relatively steeper for focal males in heterogenous versus homogenous groups, indicating that males which more frequently mate in the last male position may have a greater competitive advantage compared to rivals when in heterogenous groups, whereas this advantage is weaker in homogenous groups. This corroborates the findings from the experiment varying female and male sizes, where we found a statistically significant interaction of treatment (Hom vs. Het) and focal male size which was driven by the steeper relationship between focal male mating order and paternity share for focal males in heterogenous versus homogenous groups (Figure 3f; *F*_1,32_ = 10.831, *p* = 0.002, Table S3).

To explore the underlying drivers of the steeper slopes for focal males in heterogeneous groups in the experiment varying male and female sizes, we split the data into data points that were either above (more matings as last male position) or below (more matings as non‐last male position) the median mating order for each group. This approach allowed us to test whether the steeper slope for focal males in heterogenous groups was due to lower paternity share of males that tended to mate in non‐last positions (i.e., index <0.5), an increase in paternity of males that tended to mate in the last position (index >0.5), or both. This analysis revealed a lower average and higher standard error in paternity for focal males that tended to mate in a non‐last position more often, compared to males that tended to mate in the last position (*HomFM* ≤ *median*: 0.693 ± 0.045, *n* = 10; *HomFM > median*: 0.785 ± 0.040, *n* = 7; *HetFM* ≤ *median*: 0.419 ± 0.081, *n* = 13; *HetFM > median*: 0.831 ± 0.063, *n* = 6, Figure 3g). Albeit weaker, a similar result was observed in the experiment varying male size (*HomM* ≤ *median*: 0.523 ± 0.067, *n* = 9; *HomFM > median*: 0.744 ± 0.046, *n* = 7; *HetFM* ≤ *median*: 0.388 ± 0.088, *n* = 8; *HetFM > median*: 0.783 ± 0.057, *n* = 7). In the experiment varying female size, focal males in heterogenous groups also had lower average and higher variance in focal male paternity, although this was observed for focal males mating more often in both first and second male positions (*HomF* ≤ *median*: 0.575 ± 0.065; *HomF > median*: 0.843 ± 0.050; *HetF* ≤ *median*: 0.444 ± 0.107; *HetF > median*: 0.627 ± 0.126). There were no statistically significant main effects of repetitive matings with same female in any of the three experiments (Table S3). These results suggest that the social composition of males and females in a mating group can strengthen the effects of male mating order on the paternity share of both large and small (focal) males.

## 4 DISCUSSION

We assembled freely mating groups where females could mate with multiple males and tested whether last‐male sperm precedence explains most of the variation in male paternity share in social groups of *D. melanogaster* and whether this effect can vary among social groups with individuals from different body sizes. We expected mating order to be the major contributor to male paternity, and large males to secure more matings and sire more offspring than small males, particularly when males of different sizes competed in the same group. Our study first confirms the expectation that last male sperm precedence explains most variance in the paternity share of male *D. melanogaster*, as previously found by Pischedda and Rice (2012). After accounting for mating order, only ca. 11%–16% of variance in focal male paternity share remained unexplained (Table 1), which is comparable to the ~8.6% in Pischedda and Rice (2012), even though the methods of variance decomposition between the studies differed. Our work corroborates the strong role of male mating order in determining male paternity and shows that this remains true, to varying degrees, across social groups with individuals of varying sizes. Conversely, our data also showed that neither small nor large males benefited substantially from repetitive matings with same females, as repetitive matings had only minor contributions to male paternity share. We partially confirmed that large males tend to have an advantage over small males when competing for large females both in terms of male mating order and repetitive matings with same females (Figure 2e,h), but not when females or both females and males varied in size (heterogenous groups; Figure 2f,i). However, we also found that the steepness of the relationship between male mating order and paternity increased for both small and large males when in heterogenous male groups irrespective of the size of females (Figure 3f), raising the possibility that heterogenous groups accentuate the importance of male mating order for male paternity share in species with last male sperm precedence.

Our data revealed that male mating order and paternity were more tightly correlated in groups with a mixture of body sizes (i.e., heterogenous groups). This effect was driven by an overall decrease in male paternity for males that tended to mate more often in non‐last positions. For instance, in the experiment varying male size, small focal males in Het social groups had lower average and higher variance in paternity share than males that tended to mate more in non‐last positions compared with similarly small focal males in Hom groups. Interestingly, in the experiment varying female and male sizes, this effect was even more pronounced (Figure 3f), highlighting that social group composition and size heterogeneity of female body sizes accentuated the effects observed in male social heterogenous groups (Figure 3g). One possibility for this effect is that the changes in the average and variance in paternity share are driven by a combination of differences in male ejaculate allocation and/or cryptic female choice, which are known factors that skew paternity and influence both average and variance male reproductive success (Birkhead & Pizzari, 2002; Parker & Pizzari, 2010). *Drosophila melanogaster* males are known to partition their ejaculate, and females have been shown to display cryptic choice (Churchill et al., 2020; Hopkins et al., 2019; Lüpold et al., 2011; Pitnick & Markow, 1994; Sirot et al., 2011; Sirot & Wolfner, 2015). This might reflect perceived differences in polyandry—although counteracting that idea we previously found no evidence of differences in polyandry levels between homogenous and heterogenous groups (Morimoto et al., 2016). Differential ejaculate allocation and cryptic female choice are widespread and important factors determining fitness across species (Parker & Pizzari, 2010) including insects (Edvardsson & Göran, 2000; Rönn et al., 2008), mice (Firman & Simmons, 2015; Ramm et al., 2015; Ramm & Stockley, 2007) and birds (Løvlie et al., 2013; Nicholls et al., 2001; Pizzari et al., 2003). Our data does not allow us to test these factors directly and this remains an important avenue for future research.

Our data suggests that repetitive mating has only minor contributions to the variance in male paternity share. This is likely because with each mating, females are likely to receive more sperm than their storage capacity and therefore repetitive matings by the same male within short intervals are unlikely to increase the number of a male's sperm stored (although repetitive matings can have effects other than increasing sperm numbers, such as increasing the transfer of seminal fluid proteins involved in oviposition; Manier et al., 2010, 2013; Sirot et al., 2011). Therefore, in general, last male sperm precedence skews male paternity above and beyond any benefits of increased sperm representation through repetitive matings (Pischedda & Rice, 2012). However, this effect may depend on polyandry levels of the group. A recent study has shown that, when polyandry levels are high, males may benefit relatively more from mating repetitively with the same female than from last male sperm precedence, due to an increase over time in the proportional representation of their ejaculates relative to the ejaculate of their rivals (Carleial et al., 2020). Furthermore, the benefits of last male precedence decrease when males mate with thrice mated females. This reduction in the benefits of last male sperm precedence is negatively associated with the interval between matings, whereby short remating intervals lead to lower paternity bias from last male sperm precedence (Laturney et al., 2018). Likewise, increasing the number of mates for female harlequin beetle‐riding pseudoscorpions *Cordylochernes scorpioides* eliminates last male sperm precedence (Zeh & Zeh, 1994). Similarly, decreasing the time between matings can also reduce the strength of last male sperm precedence in the red flour beetle *Tribolium castaneum* (Arnaud et al., 2001). Therefore, it is likely that polyandry levels modulate the relative contributions of last male sperm precedence and repetitive mating with same females to the variance in male paternity.

It is important to mention that in our experiment varying male size, both male mating order and repetitive matings with same females contributed to a sizable portion of the explained variance in paternity. Moreover, the relationship between focal male mating order and paternity share was stronger when large focal males were accompanied by large females (LM:LF) compared with when small focal males were accompanied by large females (SM:LF). In fact, the strength of the relationship in the homogenous large group LM:LF were similar to that observed in the heterogenous groups (i.e., HetM:LF for both small and large focal males; Figure 3e). There was no evidence that females in the LM:LF group were more polyandrous than in the other treatments in the same experiment (Morimoto et al., 2016) and thus the reasons for these effects remain unknown. For instance, we cannot rule out stochastic effects from our relatively low sample size. Moreover, males in this treatment could also have partitioned ejaculate in ways that made repetitive mating an important factor, in addition to last male sperm precedence, in determining paternity. In fact, this could underpin our previous findings, in the same treatment, where the strength of the female Bateman gradient (i.e., slope of female mating success on reproductive success) was steeper than predicted by theory (Morimoto et al., 2016).

Larval density underpins organism‐wide physiological and behavioural responses above and beyond body size alone (Morimoto, Wenzel, et al., 2022). For example, in *Drosophila*, larval density can lead to hormesis‐like responses that increase survival (Henry et al., 2018; Lushchak et al., 2019). High larval density can be a signal of future competition levels and therefore, can also modulate mating strategies. Male *D*. *melanogaster* from high larval density have relatively larger reproductive organs, and invest proportionately more seminal fluid per mating than large males (De Nardo et al., 2021; Morimoto, Barcellos, et al., 2022; Wigby et al., 2015) (see also McGraw et al., 2007). Thus, the mechanisms that led to differences in last male precedence found in this study, and its relative importance for male fitness, are likely multifaceted, including both body size effects and plasticity related to the perception of competition. Parsing these mechanisms is experimentally challenging, and lies beyond the scope of the current study. One avenue for future studies would be to manipulate both larval and adult densities and investigate the extent to which the physiological and behavioural responses of large and small individuals are adaptive.

## 5 CONCLUSION

Body size is a known sexually selected trait in invertebrates and vertebrates (Halliday & Verrell, 1986; Janicke & Fromonteil, 2021; Suárez‐Tovar et al., 2019). Our findings show that within group variation in male and female body size composition modulate the relative importance of male mating order in biasing male paternity share and hence, post‐copulatory sexual selection. Specifically, our results suggest that—while males that mate in the last position sire more offspring as expected—this advantage can vary depending on the male body size composition of groups. This indicates that the impact of mating order may be stronger in nature when male rivals vary in size compared to expectations from laboratory settings where male size is experimentally controlled. Moreover, our results suggest that post–copulatory sexual selection on male body size is also dependent on female body size, because small males are likely less able to overcome the mating resistance of larger, already mated females. Given post‐copulatory sexual selection and female polyandry are widespread in nature (Taylor et al., 2014), future research should aim to link the ecological factors that modulate variation in male and female phenotypes within groups and its consequences for post‐copulatory processes. Such approaches will advance our understanding the socio‐ecology of sexual selection across the animal kingdom.

## AUTHOR CONTRIBUTIONS

**Juliano Morimoto:** Conceptualization (equal); data curation (lead); formal analysis (lead); funding acquisition (equal); investigation (lead); methodology (equal); visualization (lead); writing – original draft (lead); writing – review and editing (equal). **Grant C. McDonald:** Data curation (supporting); formal analysis (supporting); validation (supporting); visualization (supporting); writing – original draft (supporting); writing – review and editing (equal). **Stuart Wigby:** Conceptualization (equal); data curation (supporting); formal analysis (supporting); funding acquisition (lead); investigation (equal); methodology (equal); project administration (lead); supervision (lead); visualization (supporting); writing – original draft (supporting); writing – review and editing (equal).

## CONFLICT OF INTEREST STATEMENT

The authors have no conflict of interests to declare.

### PEER REVIEW

The peer review history for this article is available at https://www.webofscience.com/api/gateway/wos/peer‐review/10.1111/jeb.14191.

## Supporting information


**Table S1.** Pearson’s correlation between focal male mating order and repetitive matings.

**Table S2.** Effects of focal male size and social groups composition on focal male mating order and repetitive matings. Bold—*p* < 0.05.

**Table S3.** The strength of the relationship between focal male mating order, repetitive matings, and paternity share with multiple mated females. Bold—*p* < 0.05.

## Data Availability

The raw data is available in Dryad: https://doi.org/10.5061/dryad.tb2rbp05t.
